# Unexpected effects of the MIP‐Cre^ER^ transgene and tamoxifen on *β*‐cell growth in C57Bl6/J male mice

**DOI:** 10.14814/phy2.12863

**Published:** 2016-09-26

**Authors:** Bethany A. Carboneau, Thao D. V. Le, Jennifer C. Dunn, Maureen Gannon

**Affiliations:** ^1^ Department of Molecular Physiology and BiophysicsVanderbilt UniversityNashvilleTennessee; ^2^ Program in Developmental BiologyVanderbilt UniversityNashvilleTennessee; ^3^ Department of MedicineVanderbilt University Medical CenterNashvilleTennessee; ^4^ Department of Veterans AffairsTennessee Valley Health AuthorityNashvilleTennessee; ^5^ Department of Cell and Developmental BiologyVanderbilt UniversityNashvilleTennessee

**Keywords:** Human growth hormone, tamoxifen, *β*‐cell mass, *β*‐cell proliferation

## Abstract

Transgenic mouse models have been fundamental in the discovery of factors that regulate *β*‐cell development, mass, and function. Several groups have recently shown that some of these models display previously uncharacterized phenotypes due to the transgenic system itself. These include impaired islet function and increased *β*‐cell mass due to the presence of a human growth hormone (hGH) minigene as well as impaired *β*‐cell proliferation in response to tamoxifen (TM) administration. We aimed to determine how these systems impact *β*‐cell mass and proliferation during high fat diet (HFD). To this end, we utilized C57Bl6/J male MIP‐Cre^ER^ mice, which are known to express hGH, or wild‐type (WT) mice treated with vehicle corn oil or TM. In the absence of TM, MIP‐Cre^ER^ mice fed a chow diet have increased *β*‐cell mass due to hypertrophy, whereas replication is unchanged. Similarly, after 1 week on HFD, MIP‐Cre^ER^ mice have increased *β*‐cell mass compared to WT, and this is due to hypertrophy rather than increased proliferation. To assess the impact of TM on *β*‐cell proliferation and mass, WT mice were treated with vehicle corn oil or TM and then fed a chow diet or HFD for 3 days. We observed that TM‐treated mice have improved glucose homeostasis on chow diet but impaired *β*‐cell proliferation in response to 3 days HFD feeding. These results unveil additional complications associated with commonly used pancreas‐specific mouse models.

## Introduction

Type 2 diabetes (T2D) results from failure of the insulin‐producing *β*‐cells to compensate for increased metabolic demands, resulting in impaired glucose homeostasis and decreased *β*‐cell mass. Normally, *β*‐cells compensate for periods of increased metabolic demand, such as pregnancy or obesity, by increasing insulin secretion and expanding functional *β*‐cell mass (Golson et al. 2010). In adult rodent islets, *β*‐cell mass expansion occurs primarily by replication of existing *β*‐cells (Dor et al. 2004). Thus, a major goal in the diabetes research field is to identify factors that promote expansion of functional *β*‐cell mass, thereby reversing the disease and preventing complications.

The Cre‐*loxP* system of genetic recombination has been a powerful tool in the discovery of factors that regulate *β*‐cell development, replication, and mass expansion. Indeed, there are numerous pancreas‐specific Cre recombinase (Cre) mouse models currently available and widely used (reviewed in [Magnuson and Osipovich 2013]). Cre excises DNA regions of interest that are flanked by two *loxP* sites (34 nucleotide‐long DNA sequences), which allows for cell‐ or tissue‐specific recombination dependent on the promoter driving Cre expression. This system has been refined to incorporate temporal control of Cre activity using Cre^ERT2^ (referred to herein as Cre^ER^) for inducible recombination. Cre^ER^ is a fusion protein consisting of Cre and a mutated, tamoxifen (TM)‐responsive estrogen receptor (ERT2) that is insensitive to endogenous 17*β*‐estradiol (Feil et al. 1997; Danielian et al. 1998). In the absence of TM, Cre^ER^ is sequestered in the cytoplasm; but upon TM binding, activated Cre^ER^ enters the nucleus and mediates recombination at *loxP* sites.

Several years ago, we and others reported that some “pancreas‐specific” Cre transgenes were also expressed in key brain regions involved in food intake and energy expenditure, complicating their use in metabolic studies (Wicksteed et al. 2010). Thus, the generation of a mouse insulin promoter (MIP)‐Cre^ER^ line in which brain recombination did not occur was a critical development in the field (Tamarina et al. 2014). More recently, it has been reported that some pancreas‐specific Cre and Cre^ER^ models have unexpected phenotypes, in the absence of any *loxP*‐containing locus, due to the Cre system itself (Brouwers et al. 2014; Oropeza et al. 2015; Yuchi et al. 2015). These issues include changes in glucose tolerance, *Glut2* expression, and *β*‐cell mass due to the presence of a human growth hormone (hGH) minigene in the transgene construct (Brouwers et al. 2014; Oropeza et al. 2015). The hGH minigene was incorporated in the design of several transgenes, including MIP‐Cre^ER^, in order to enhance mRNA stability and expression levels (Palmiter et al. 1991; Orban et al. 1992; Tamarina et al. 2014). Islets isolated from mice expressing Cre transgenes that contain hGH were found to express *hGH* mRNA and protein (Brouwers et al. 2014; Oropeza et al. 2015). Further characterization of MIP‐Cre^ER^ mice revealed that the presence of hGH alters pancreatic insulin content and *β*‐cell mass in the presence of TM, as well as protects against hyperglycemia induced by combined high fat‐high sugar diet and streptozotocin (STZ) treatment independent of TM (Oropeza et al. 2015). Similar observations were also made in a different strain of mice that contains hGH in the transgenic construct: the Pdx1‐Cre^late^ mouse (Brouwers et al. 2014). Additionally, it was recently reported that TM, in the absence of Cre^ER^, impairs compensatory *β*‐cell proliferation in the settings of partial duct ligation (PDL), pregnancy, and during development (Yuchi et al. 2015). Thus, the utility of these commonly used tools, as well as the validity of findings related to *β*‐cell proliferation, function, and mass, have been called into question.

Our group studies the regulation of adult *β*‐cell proliferation during periods of metabolic stress, such as high fat diet (HFD)‐induced obesity. We embarked on studies of HFD‐induced *β*‐cell mass expansion, making use of the MIP‐Cre^ER^ strain to mediate *β*‐cell‐specific recombination in adults. Therefore, we wanted to determine if *β*‐cell proliferation was altered by either the MIP‐Cre^ER^ transgene or TM during HFD in the absence of any additional genetic manipulation. To date, there are no reports on the impact of MIP‐Cre^ER^ or TM on HFD‐induced *β*‐cell proliferation. Thus, we evaluated *β*‐cell proliferation and mass in 8‐week‐old MIP‐Cre^ER^ mice in the absence of TM as well as in wild‐type (WT) mice injected with vehicle corn oil or TM and fed either chow diet or HFD. Our goal was to determine if the presence of the MIP‐Cre^ER^ transgene or TM administration alters *β*‐cell proliferation, *β*‐cell mass, and whole‐body glucose metabolism. Our results clearly demonstrate that the MIP‐Cre^ER^ transgene leads to increased *β*‐cell mass via hypertrophy, whereas TM impairs HFD‐induced *β*‐cell proliferation. The results presented here raise serious concerns about using these tools to study *β*‐cell mass dynamics.

## Materials and Methods

### Animals

Male C57BL/6J and MIP‐Cre^ER^ (Tg(Ins1‐Cre/ERT)1Lphi) (Tamarina et al. 2014) mice were purchased from Jackson Laboratory (Bar Harbor, ME). All MIP‐Cre^ER^ mice were hemizygous for the Cre driver allele and were maintained on a C57Bl/6J background. Mice were fed a chow diet (11% kcal from fat, Lab Diet 5LJ5; Purina, St. Louis, MO). For HFD studies, 8‐week‐old mice were fed a HFD (60% kcal from fat; BioServ F3282, Frenchtown, NJ) for 3 days or 1 week.

Tamoxifen (TM, T5648; Sigma‐Aldrich, St. Louis, MO) was dissolved in filtered corn oil (C8267; Sigma‐Aldrich, St. Louis, MO) to make a solution of 25 mg/ml. 6‐week‐old C57Bl/6J mice were administered 5 mg TM (200 *μ*L volume) or vehicle corn oil by subcutaneous injections for a total of 3 doses over a 5‐day period. Injections were sealed with Vetbond tissue adhesive to prevent leakage. A 1‐week washout period after injections was provided prior to any additional measurements.

Mice were housed in a 12‐h light/dark cycle and given ad libitum access to food and water. The Vanderbilt University Institutional Animal Care and Use Committee approved all mouse studies.

### Intraperitoneal glucose tolerance tests

Intraperitoneal glucose tolerance tests (IP‐GTT) were performed as previously described (Henley et al. 2012) following a 16‐h fast in 8‐week‐old mice or after HFD feeding.

### Tissue preparation and histology

Pancreata were dissected, fixed, and processed as previously described (Golson et al. 2010). Antibodies used included guinea pig anti‐insulin (1:500; Dako, Carpinteria, CA), rabbit anti‐Ki67 (1:500; AbCam, Cambridge, MA), rabbit anti‐Cre (1:2000, Millipore, San Diego, CA), Cy2‐conjugated anti‐guinea pig IgG (1:400; Jackson ImmunoResearch Laboratories, West Grove, PA), Cy3‐conjugated anti‐rabbit IgG (1:400; Jackson ImmunoResearch Laboratories, West Grove, PA), and horseradish peroxidase‐conjugated anti‐guinea pig IgG (1:400; Jackson ImmunoResearch Laboratories, West Grove, PA). Nuclei were visualized with 4′,6′‐diamidino‐2‐phenylindole (DAPI, 1 *μ*g/mL; Molecular Probes, Grand Island, NY). The antigen retrieval used for Ki67 immunolabeling consisted of microwaving slides for 14 min on high power in 10 mmol/L sodium citrate buffer pH 6.0. The antigen retrieval required for Cre immunostaining consisted of microwaving slides on high for 1 minute followed by 7.5 min at 10% power in 1× Tris‐EGTA buffer (TEG) pH 9.0.

### *β*‐cell mass

About 10 to 12 slides spaced 250 *μ*m apart (1–2% of the total pancreas) were immunolabeled for insulin, visualized using the DAB Peroxidase Substrate Kit (Vector Laboratories, Burlingame, CA), and counterstained with eosin. *β*‐cell mass was quantified as described (Golson et al. 2014). Images were acquired using a ScanScope CS slide scanner (Aperio Technologies, Inc., Vista, CA) and were processed using ImageScope Software (Aperio Technologies, Inc., Vista, CA). *β*‐cell mass was calculated by measuring the ratio of insulin‐positive area to total pancreas area of all sections scanned and multiplying by the pancreatic wet weight.

### *β*‐cell proliferation

Five slides spaced at least 250 *μ*m apart per animal were immunolabeled for insulin and Ki67. Images were obtained using a ScanScope FL slide scanner (Aperio Technologies, Inc., Vista, CA). A minimum of 5000 cells were manually counted using Metamorph 6.1 software (Molecular Devices, Sunnyvale, CA). The percentage of proliferating *β*‐cells was determined by dividing the total number of Ki67‐insulin double‐positive cells by the total number of insulin‐positive cells.

### *β*‐cell size and number

Five slides spaced at least 250 *μ*m apart per animal were immunolabeled for insulin. Images were acquired using the methods described for *β*‐cell proliferation. Average *β*‐cell size was determined by dividing the insulin‐positive area by the total number of *β*‐cells. A minimum of 100 islets per animal was assessed. *β*‐cell number was measured as the total number of *β*‐cells counted per 100 islets.

### Islet size

Sections were imaged and analyzed for insulin as described in *β*‐cell mass. Insulin+ clusters were counted and binned by size (<8 or ≥8 cells).

### Statistics

All results are expressed as mean ± SEM. Statistical significance was calculated in GraphPad Prism Version 6.0d (GraphPad Software, Inc., La Jolla, CA) using a two‐way ANOVA and Tukey post hoc analysis for IP‐GTT data. All other data were analyzed via Student's *t‐*test. *P* values of ≤0.05 were considered significant.

## Results

### Chow‐fed MIP‐Cre^ER^ mice have a tamoxifen‐independent increase in *β*‐cell mass due to hypertrophy

A recent study found that chow‐fed MIP‐Cre^ER^ mice treated with TM have a strong trend toward increased *β*‐cell mass (*P* = 0.051), likely due to the presence of hGH in the transgenic construct (Oropeza et al. 2015). This is similar to the findings of another commonly used model that also contains the hGH minigene, the Pdx1‐Cre^late^ strain (Brouwers et al. 2014). Pdx1‐Cre^late^ mice have significantly increased *β*‐cell mass at 24 weeks of age (Brouwers et al. 2014). However, neither group determined the mechanism for increased *β*‐cell mass. Since a major focus of our group is regulation of adult *β*‐cell proliferation, we wanted to determine whether this parameter could be affected by the hGH‐containing MIP‐Cre^ER^ transgene.

**Figure 1:**
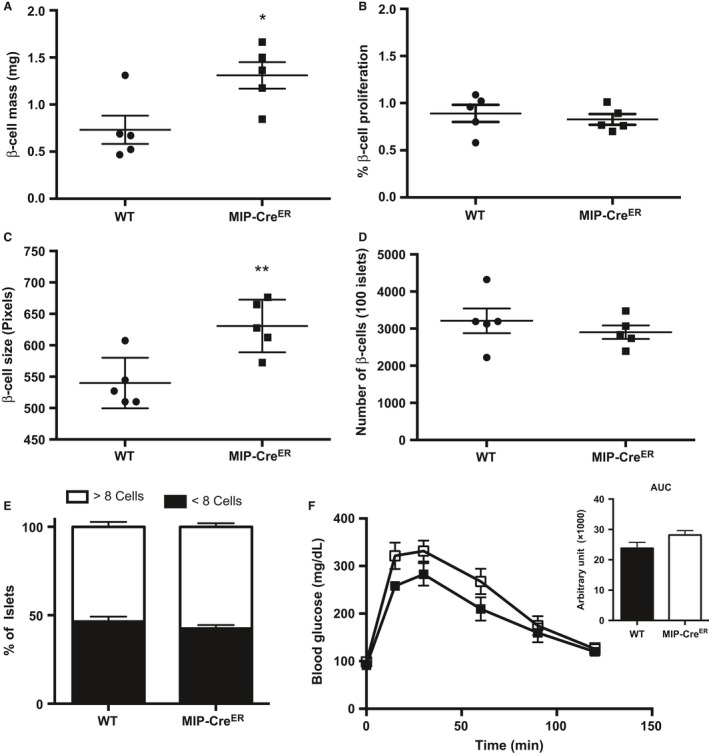
Chow‐fed MIP‐Cre^ER^ mice have enhanced *β*‐cell mass due to increased *β*‐cell size. (A) *β*‐cell mass, (B) *β*‐cell proliferation, (C) *β*‐cell size, (D) *β*‐cell number, (E) percentage of small insulin+ clusters, and (F) Intraperitoneal glucose tolerance tests (IP‐GTT) and area under the curve (AUC) of IP‐GTT for chow‐fed 8‐week‐old WT (*n* = 5) or MIP‐Cre^ER^ (*n* = 5) male mice. Black boxes represent WT mice; open boxes represent MIP‐Cre^ER^ mice. Data are shown as means ± SEM. *P* values were calculated using Student's *t‐*test for (A–C), (E) and two‐way ANOVA followed by Tukey post hoc analysis for D. *P = 0.02 in (A); **P = 0.0081 in (C).

We measured *β*‐cell mass in 8‐week‐old untreated MIP‐Cre^ER^ and WT male mice fed a chow diet. Compared to WT mice, MIP‐Cre^ER^ mice had significantly increased *β*‐cell mass (Fig. 1A). We then assessed *β*‐cell proliferation and *β*‐cell size to determine if either of these compensatory mechanisms could explain the increase in *β*‐cell mass in MIP‐Cre^ER^ mice. While *β*‐cell proliferation was similar between WT and MIP‐Cre^ER^ mice (Fig. 1B), individual *β*‐cell size was significantly increased in MIP‐Cre^ER^ mice (Fig. 1C). However, to determine whether the increase in *β*‐cell mass in MIP‐Cre^ER^ mice could be explained by an increase in proliferation earlier than 8 weeks of age, we evaluated *β*‐cell number in each genotype. No differences in total *β*‐cell number were observed between chow‐fed MIP‐Cre^ER^ mice and WT mice (Fig. 1D), suggesting that the increase in *β*‐cell mass in MIP‐Cre^ER^ mice at 8 weeks of age is not due to an earlier increase in proliferation. We also explored the possibility that an increase in islet neogenesis could account for the increase in *β*‐cell mass in MIP‐Cre^ER^ mice. As a surrogate for neogenesis, we quantified the number of small insulin+ clusters (<8 cells). No change in number of small islet clusters was observed (Fig. 1E), suggesting that neogenesis does not contribute to the increase in *β*‐cell mass. However, in the absence of a lineage tracer, we cannot completely rule this out. Thus, we conclude that MIP‐Cre^ER^ mice have increased *β*‐cell mass due to individual cell hypertrophy and not due to changes in *β*‐cell proliferation. To our knowledge, this is the first report showing that the TM‐independent increase in *β*‐cell mass observed in MIP‐Cre^ER^ mice is due to *β*‐cell hypertrophy rather than proliferation.

We also assessed whole‐body glucose homeostasis as the production of Cre recombinase in *β*‐cells has led to impaired glucose homeostasis in other models in the absence of TM (Lee et al. 2006). Consistent with a recent report (Oropeza et al. 2015), chow‐fed untreated MIP‐Cre^ER^ mice showed no significant difference in glucose clearance as measured by intraperitoneal glucose tolerance tests (IP‐GTT) (Fig. 1F).

### β‐cell proliferation is affected similarly in HFD‐fed WT and MIP‐Cre^ER^ mice

**Figure 2:**
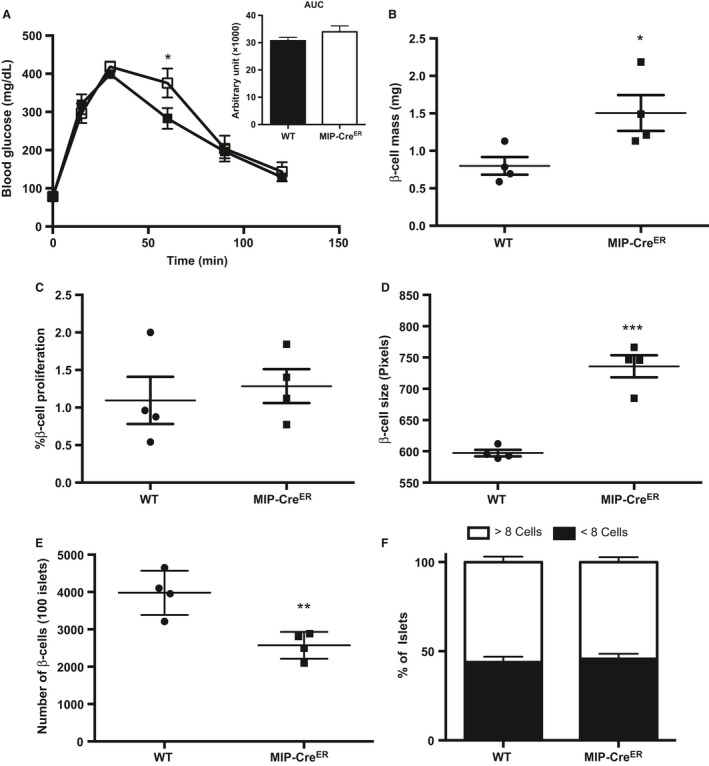
Wild‐type (WT) and MIP‐Cre^ER^ mice show similar levels of *β*‐cell proliferation in response to 1 week high fat diet (HFD) feeding. (A) Intraperitoneal glucose tolerance tests (IP‐GTT) and area under the curve (AUC) (WT *n* = 6, MIP‐Cre^ER^ *n* = 3), (B) *β*‐cell mass, (C) *β*‐cell proliferation, (D) *β*‐cell size, (E) *β*‐cell number, and (F) percentage of small insulin+ clusters for 8‐week‐old WT or MIP‐Cre^ER^ male mice placed on a HFD for 1 week. Black boxes represent WT mice; open boxes represent MIP‐Cre^ER^ mice. Data are shown as means ± SEM. *P* values were calculated using Student's *t‐*test for (B–D) and two‐way ANOVA followed by Tukey post hoc analysis for (A). *P < 0.05 in (A); *P = 0.0385 in (B); and ***P = 0.0003 in (D); **P = 0.0067 in (E).

When *β*‐cell compensatory mechanisms are operating normally, *β*‐cell mass is increased during obesity in both humans and rodents in response to increases in metabolic demand (Butler et al. 2003; Hull et al. 2005; Tschen et al. 2009). It is well documented that in rodents, this increase in *β*‐cell mass during obesity occurs via increased *β*‐cell proliferation (reviewed in [Linnemann et al. 2014]). Although MIP‐Cre^ER^ mice on a chow diet did not show changes in *β*‐cell proliferation compared to WT mice, we wanted to investigate if HFD‐induced replication was altered due to the presence of the hGH minigene in this strain. Eight‐week‐old WT and MIP‐Cre^ER^ mice were placed on a HFD for 1 week; a time point that corresponds to robust increases in proliferation (Stamateris et al. 2013; Mosser et al. 2015). Whole‐body glucose homeostasis was unchanged between WT and MIP‐Cre^ER^ mice following HFD‐feeding as measured by IP‐GTT (Fig. 2A). Furthermore, HFD‐fed MIP‐Cre^ER^ animals have increased *β*‐cell mass and *β*‐cell size compared to WT (Fig. 2B, D), similar to chow‐fed mice. There was no difference in *β*‐cell proliferation in response to 1‐week HFD feeding between WT and MIP‐Cre^ER^ (Fig. 2C). The increased *β*‐cell mass can be explained again by hypertrophy, rather than proliferation or neogenesis, as we observed a decrease in the number of *β*‐cells in MIP‐Cre^ER^ mice on HFD (Fig. 2E), and no difference in the number of small insulin+ clusters (Fig. 2F). Collectively, these data suggest that neither the presence of hGH nor the MIP‐Cre^ER^ transgene itself affects *β*‐cell proliferation during metabolic stress, but that one or both affects *β*‐cell growth.

### Tamoxifen inhibits HFD‐induced *β*‐cell proliferation

**Figure 3:**
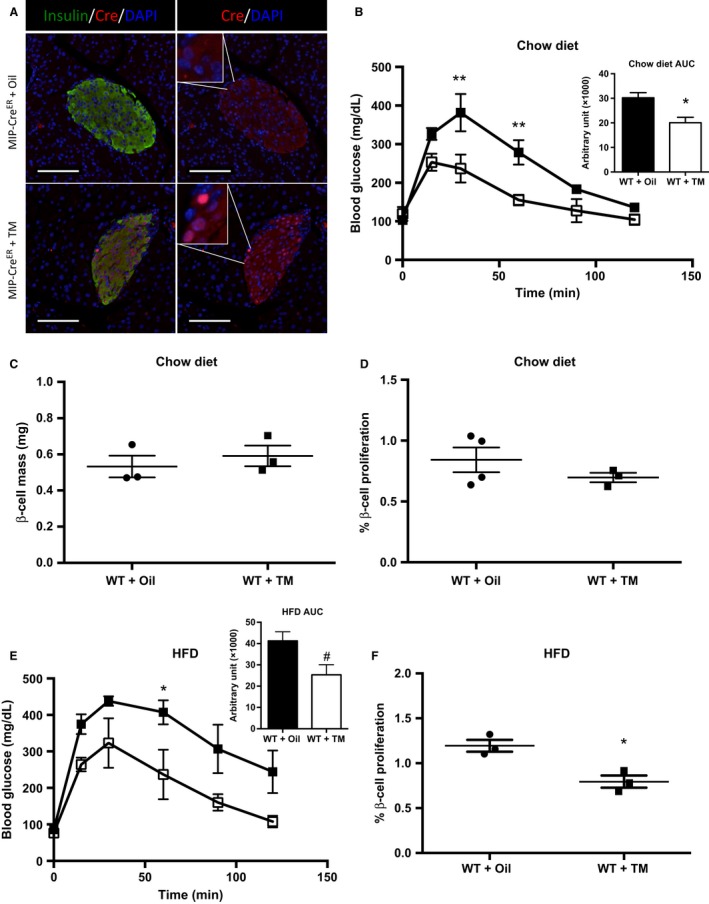
Tamoxifen (TM) inhibits *β*‐cell proliferation after 3 days high fat diet (HFD) feeding in Wild‐type (WT) mice. (A) Immunolabeling of Cre localization in MIP‐Cre^ER^ mice injected with corn oil (top panels) or TM (bottom panels). In the absence of TM, Cre displays cytoplasmic localization (top panels). Upon TM treatment, Cre immunolabeling can be visualized in the nuclei, as marked by DAPI (bottom panels). Scale bars represent 100 *μ*m. (B) Intraperitoneal glucose tolerance tests (IP‐GTT) and area under the curve (AUC) (WT + oil *n* = 3, WT + TM *n* = 3), (C) *β*‐cell mass, and (D) *β*‐cell proliferation for chow‐fed WT mice injected with corn oil or TM at 6 weeks of age and analyzed at 8 weeks of age. (E) IP‐GTT and AUC (WT + oil n = 3, WT + TM *n* = 3) and (F) *β*‐cell proliferation for WT mice placed on HFD for 3 days following the washout period. Black boxes represent WT + Oil‐treated mice; open boxes represent WT + TM mice. Data are shown as means ± SEM. *P* values were calculated using Student's *t‐*test for (C), (D), and (F) and two‐way ANOVA followed by Tukey post hoc analysis for (B) and (E). *P = 0.0291, **P < 0.01 in (B); ^#^ *P* = 0.0696, *P < 0.05 in (E); and *P = 0.0133 in (F).

The above studies were performed in MIP‐Cre^ER^ mice in which Cre^ER^ had not been activated by TM injection and was retained in the cytoplasm (Fig. 3A, top panels). Upon TM injection, MIP‐Cre^ER^ undergoes nuclear translocation (Fig. 3A, bottom panels). Recently, TM was reported to impair *β*‐cell proliferation during PDL, pregnancy, and development in the absence of any genetic manipulation (Yuchi et al. 2015). However, the effects of TM on HFD‐induced *β*‐cell proliferation are unknown. As TM is required for activation of MIP‐Cre^ER^, we sought to determine if the presence of TM, in the absence of Cre, affects basal and/or HFD‐induced *β*‐cell proliferation.

We first measured glucose homeostasis and *β*‐cell proliferation in chow‐fed mice. For these experiments, 6‐week‐old WT male mice were injected with corn oil (vehicle) or TM dissolved in corn oil (3 × 5 mg injections over 5 days). Following a 1‐week washout period, IP‐GTTs were performed. WT mice injected with TM have significantly improved glucose homeostasis compared to oil‐injected mice (Fig. 3B). No significant effect of TM on *β*‐cell mass or *β*‐cell proliferation was observed (Fig. 3C and D). These findings are similar to previously published data in nonpregnant female mice (Yuchi et al. 2015).

We then investigated whether TM impacts compensatory *β*‐cell proliferation. To address this question, WT male mice were placed on a HFD for 3 days following the 1‐week washout period mentioned above. We have previously published that 3 days of HFD feeding is the peak in HFD‐induced *β*‐cell proliferation (Mosser et al. 2015). There was a trend for improved glucose homeostasis in WT mice injected with TM (Fig. 3E). *β*‐Cell proliferation was blunted in TM‐treated mice compared to oil‐injected mice following 3 days of HFD (Fig. 3F). Our data add to the growing body of literature showing deleterious effects of TM on *β*‐cell proliferation in response to different stimuli. Here, we show that HFD‐induced proliferation is blunted with TM treatment, similar to what others have shown for PDL, pregnancy, and development (Yuchi et al. 2015).

## Discussion

In this study, we confirm and expand upon recent findings characterizing commonly used mouse models in the islet field (Oropeza et al. 2015; Yuchi et al. 2015). We show that MIP‐Cre^ER^ mice do not have impairments in whole‐body glucose homeostasis yet have increased *β*‐cell mass in the absence of TM. Our report demonstrates for the first time that the increase in *β*‐cell mass can be explained by hypertrophy and is not due to changes in replication. We further show that the presence of the MIP‐Cre^ER^ transgene does not alter *β*‐cell proliferation during HFD conditions. We also observe that TM administration improves glucose homeostasis in chow‐fed mice but impairs *β*‐cell proliferation after HFD feeding. Our findings reveal several new concerns associated with transgenic mouse models regularly used to study *β*‐cell development, function, and mass dynamics.

Growth hormones (GH) can act on the *β*‐cell by activating prolactin receptors (PRLRs) (Goffin et al. 1996) and exert similar effects as prolactin (PRL) signaling (Parsons et al. 1995). Binding of PRL, placental lactogen (PL), and GH to PRLR activates Janus kinase (JAK2) signaling, resulting in phosphorylation and activation of the transcription factor signal transducer and activation of transcription 5 (STAT5). During pregnancy, PRL and PL induce production of serotonin and increase *β*‐cell proliferation, thus expanding *β*‐cell mass to compensate for insulin resistance (Pasek and Gannon 2013). It has recently been reported that hGH expressed from Cre transgenes can contribute to pregnancy‐like phenotypes (Brouwers et al. 2014; Oropeza et al. 2015). Specifically, MIP‐Cre^ER^ mice have increased expression of *tryptophan hydroxylase 1* (*Tph1*) and *Tph2*, the enzymes responsible for serotonin production in addition to increased serotonin (Oropeza et al. 2015). These effects have been observed in different genetic backgrounds of mice, including the C57Bl6/J, CD1, and BTBR strains (Brouwers et al. 2014; Baan et al. 2015; Oropeza et al. 2015). Here, we show that, in addition to the pregnancy‐associated phenotypes previously reported, MIP‐Cre^ER^ mice have increased individual *β*‐cell size resulting in significantly increased *β*‐cell mass. hGH has been shown to augment *β*‐cell proliferation both in vivo (Baan et al. 2015) and ex vivo in cultured islets (Nielsen et al. 1989). However, in our study, the presence of the hGH in the MIP‐Cre^ER^ transgene does not result in altered *β*‐cell replication. These differences may be due to the genetic makeup of transgenes used in the different studies (Tamarina et al. 2014; Baan et al. 2015). Consistent with our findings, *β*‐cell proliferation was not different between untreated MIP‐Cre^ER^ and WT mice placed on a high fat‐high sugar diet and treated with STZ (Oropeza et al. 2015).

In addition to the effects of hGH on *β*‐cell dynamics, the consequences of TM have recently been brought to light. TM is a synthetic antagonist that binds a mutated ER that is insensitive to circulating estrogens (Jaisser 2000). Estrogen and ER signaling are important in *β*‐cell function, survival, and replication (Yuchi et al. 2015), yet the effect of TM on *β*‐cell proliferation was previously unknown. TM administration, in the absence of Cre, resulted in decreased *β*‐cell proliferation following PDL. This was phenocopied in ER*α* ^−/−^ mice, demonstrating the importance of ER signaling in promoting *β*‐cell proliferation (Yuchi et al. 2015). This group further reported that TM impairs the induction of *β*‐cell proliferation observed during embryonic development and pregnancy (Yuchi et al. 2015). We have now shown that TM administration also blunts the proliferative capacity of *β*‐cells in response to short‐term HFD feeding.

Wild‐type mice injected with oil have a 1.42‐fold increase in proliferation in response to HFD, whereas TM‐treated mice only display a 1.13‐fold change. The fold change in proliferation observed here in response to 3 days HFD is less than what we have previously published (Mosser et al. 2015). This can be explained, in part, by the stress induced on the mice by the subcutaneous injections of oil or TM. In our previous publication, uninjected WT mice were placed on a HFD (Mosser et al. 2015). In this study, mice received 3 injections of oil or TM prior to HFD feeding. Based on these differences, it is difficult to compare the levels of proliferation between the two studies directly.

To our surprise, the impaired proliferation in TM‐injected mice was accompanied by improved glucose homeostasis. Steroid hormones and ER modulators, such as TM, have been shown to alter food consumption and appetite (Wallen et al. 2001). Male mice treated with TM were found to consume less food than control‐treated mice, resulting in less weight gain over a 28‐day period (Larosche et al. 2007). Decreased food consumption in TM‐treated mice may explain the improved glucose homeostasis observed in our study. Higher doses of TM, similar to that used in this study, can have long‐term toxic effects in mice (Reinert et al. 2012). It remains unknown how lower doses of TM, a longer washout period, route of administration (gavage, subcutaneous, or IP) or mouse strain may impact *β*‐cell proliferation during HFD. On the basis of previous results with 3 × 1 mg TM treatment (Yuchi et al. 2015), we speculate that lower doses of TM would also inhibit HFD‐induced replication, however, individual investigators may want to determine this directly. TM administered subcutaneously is present in the system for up to 4 weeks post injection (Reinert et al. 2012). Therefore, a washout period of longer than 4 weeks may help avoid the TM‐induced impaired proliferation observed here and by Yuchi and colleagues; however, this needs to be empirically tested. Interestingly, the studies by Yuchi et al. were performed in the Balb‐C strain of mice. Taken together, our data and theirs suggest that different strains of mice will have similar responses to TM treatment with regard to effects on *β*‐cell proliferation.

In conclusion, our data unveil additional unexpected phenotypes of commonly used models for studies on *β*‐cell mass expansion, underscoring the importance of proper controls for utilization and interpretation of data related to *β*‐cell function, mass, and proliferation. In particular, inclusion of mice expressing only MIP‐Cre^ER^ or Cre^ER^ mice treated with TM without further genetic manipulation will be necessary as control groups, particularly for studies related to *β*‐cell dynamics. It should be noted that these studies were performed on a C57Bl6/J background and the observed effects may differ based on genetic background and should be tested by the investigator.

## Conflict of Interest

None declared.
